# Depletion of B cell-activating factor attenuates hepatic fat accumulation in a murine model of nonalcoholic fatty liver disease

**DOI:** 10.1038/s41598-018-37403-y

**Published:** 2019-01-30

**Authors:** Yoshiko Nakamura, Masanori Abe, Keitarou Kawasaki, Teruki Miyake, Takao Watanabe, Osamu Yoshida, Masashi Hirooka, Bunzo Matsuura, Yoichi Hiasa

**Affiliations:** Department of Gastroenterology and Metabology, Ehime University Graduate School of Medicine, Ehime, 791-0295 Japan

## Abstract

Obesity-induced adipose-tissue dysfunction is a critical contributor to the pathogenesis of nonalcoholic fatty liver disease (NAFLD). B cell-activating factor (BAFF) is an adipokine related to impaired insulin sensitivity, and the serum BAFF concentration is associated with NAFLD severity. In this study, we aimed to determine the direct *in vivo* role of BAFF in the development of insulin resistance, adipocyte dysfunction, and hepatic steatosis using *BAFF*^−/−^ mice fed a high-fat diet (HFD). HFD-fed *BAFF*^−/−^ mice exhibited significantly improved insulin sensitivity despite their increased weight gain and adiposity relative to HFD-fed wild-type mice. Moreover, inflammation, especially the accumulation of CD11c^+^ adipose-tissue macrophages, and fibrosis of epididymal adipose tissue were reduced, contributing to healthy adipose-tissue expansion in obese *BAFF*^−/−^ mice. In line with metabolically healthy obesity, hepatic steatosis also decreased, and we observed attenuated *de novo* lipogenesis in both the livers and hepatocytes of *BAFF*^−/−^ mice. Our data revealed that BAFF serves as a potential stimulator of unhealthy adipose-tissue expansion by triggering inflammation and fibrosis and ultimately leading to enhanced insulin resistance and NAFLD. Therefore, these results suggest that BAFF is a promising target for diabetes and NAFLD treatment.

## Introduction

Nonalcoholic fatty liver disease (NAFLD) is currently the most common subtype of chronic liver disease worldwide^1^ and is closely related to insulin resistance and metabolic risk factors. NAFLD represents a hepatic manifestation of metabolic syndrome^2^, and numerous pathogenic symptoms, such as insulin resistance, inflammation, enhanced oxidative stress, and mitochondrial dysfunction, are implicated in NAFLD development.

A fundamental function of adipose tissue involves the storage of excess energy in the form of triglycerides (TGs). Promoting healthy adipose-tissue expansion and better lipid storage in visceral adipose tissue (VAT) is crucial to maintaining glucose homeostasis and insulin sensitivity. Recent studies have suggested that increased adipose-tissue inflammation triggers lipolysis and tissue fibrosis, thereby accelerating the release of free fatty acids (FFAs) from adipose tissue^3^ and resulting in its ectopic accumulation in non-adipose tissue such as the liver^4^. The resulting lipotoxicity subsequently initiates a variety of metabolic disorders.

Obesity-associated inflammation is also accepted as a critical factor that initiates or exacerbates NAFLD^5^. Macrophage infiltration occurs in both adipose tissue and the liver to contribute to insulin resistance and NAFLD. Proinflammatory mediators exert direct effects on hepatocytes to increase lipid synthesis^6^ and/or act on both liver macrophages and hepatocytes to accelerate hepatic inflammation and promote hepatic steatosis. Recent reports indicate that *de novo* lipogenesis is a prominent abnormality in NAFLD and the key feature involved in progression to severe steatosis^7^. Combined with excess adipose FFA release, distinct increases in lipogenesis may contribute to obesity-related NAFLD.

B cell-activating factor (BAFF; CD257) belongs to the tumour necrosis factor (TNF)-ligand family and promotes B cell proliferation and survival, leading to increased serum immunoglobulin levels^8^. Additionally, BAFF plays an important role in the development of autoimmune diseases^8^. Previous studies have reported that BAFF is produced by mature adipocytes, as well as myeloid lineage cells and activated T cells^9,10^. Using mice with diet-induced obesity, we previously demonstrated that BAFF controls the production of adipokines and induces insulin resistance via impairment of insulin-receptor signalling *in vitro*^9^. Moreover, we found that BAFF is associated with NAFLD severity among Japanese patients^11^. These data suggest that BAFF is involved in glucose and lipid metabolism in obesity.

In an effort to better understand the impact of BAFF signalling on lipid metabolism *in vivo*, we previously investigated BAFF receptor (BAFF-R)-deficient mice^12^. Contrary to our expectation, hepatic steatosis was enhanced in obese BAFF-R-deficient mice, whereas glucose tolerance was improved and body weight was reduced relative to those of obese wild-type (WT) mice^12^. However, BAFF-R-deficient mice displayed increases in serum BAFF concentrations; therefore, there remains a lack of knowledge concerning the pathological significance of BAFF signalling in NAFLD. In this study, we aimed to determine the role of BAFF in insulin resistance, adipose-tissue dysfunction, and hepatic steatosis using high-fat diet (HFD)-fed mice. Our results demonstrated that, despite increased adiposity, BAFF deficiency ameliorated obesity-associated insulin resistance and inflammation in VAT and prevented fat accumulation in the liver. Our findings suggest that BAFF may have a potential clinically relevant impact on NAFLD pathogenesis.

## Results

### Altered lipid distribution and insulin sensitivity in *BAFF*^−/−^ mice fed an HFD

**Figure 1:**
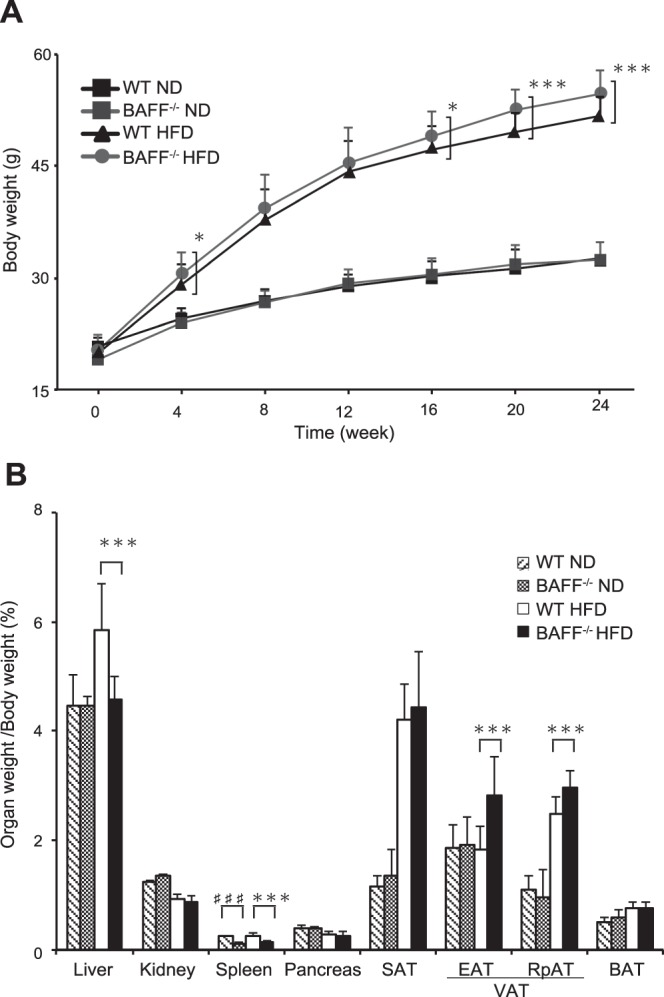
Lipid distribution in adipose tissue and liver is altered in *BAFF*^−/−^ mice fed an HFD. (**A**) Changes in body weight of *BAFF*^−/−^ and WT mice fed an ND or HFD (*n* = 25–55/group). (**B**) Organ weight [(organ weight/body weight) × 100%] in *BAFF*^−/−^ and WT mice fed an ND (*n* = 6/group) or HFD (*n* = 7/group) for 24 weeks. For all bar plots shown, data are expressed as the mean ± SD, and significance was determined by two-tailed Student’s *t* test (**A**) and Mann–Whitney *U* test (**B**). ^♯♯♯^*P* < 0.001 compared to WT-ND mice. *P < 0.05, **P < 0.01, and ***P < 0.001 compared to WT-HFD mice. BAFF, B cell-activating factor; WT, wild-type; ND, normal diet; HFD, high-fat diet; SAT, subcutaneous adipose tissue; EAT, epididymal adipose tissue; RpAT, retroperitoneal adipose tissue; VAT, visceral adipose tissue; BAT, brown adipose tissue.

We confirmed *BAFF* gene deletion in *BAFF*^−/−^ mice by serum enzyme-linked immunosorbent assay (ELISA) and detection of mRNA expression in several organs (Supplementary Fig. S1). *BAFF*^−/−^ mice showed splenic deficiencies and significantly reduced splenic weights as compared with those of WT mice (Fig. 1B).

There was no difference in body weight between normal chow diet (ND)-fed WT and *BAFF*^−/−^ mice; however, the body weight of HFD-fed *BAFF*^−/−^ mice was significantly higher than that of HFD-fed WT mice (Fig. 1A). Although VAT weight was significantly increased in HFD-fed *BAFF*^−/−^ mice as compared with that in HFD-fed WT mice, liver weight was decreased (Fig. 1B). Serum TG levels did not differ between the two HFD-fed groups (WT: 56 ± 6 mg/dL; *BAFF*^−/−^: 49 ± 12 mg/dL; *n* = 7/group; *P* = 0.25).

**Figure 2:**
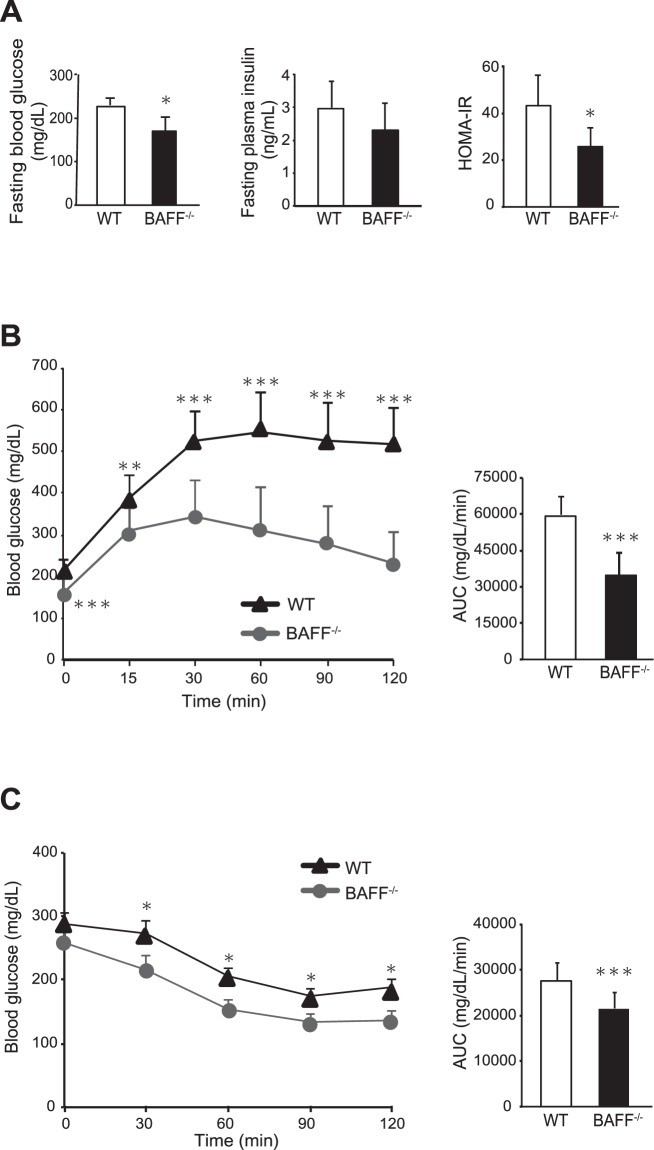
*BAFF*^−/−^ mice are protected against glucose intolerance and insulin resistance. (**A**) Fasting blood glucose, plasma insulin, and HOMA-IR values in HFD-fed *BAFF*^−/−^ and WT mice over the course of 12 weeks (*n* = 6/group). (**B**) Glucose- and (**C**) insulin-tolerance tests (left) and corresponding area under the curve (AUC) values during the tests (right) (*n* = 7–14/group). For all bar plots, data are expressed as the mean ± SD, and significance was determined by the Mann–Whitney *U* test. *P < 0.05, **P < 0.01, and ***P < 0.001. BAFF, B cell-activating factor; WT, wild-type; ND, normal diet; HFD, high-fat diet; HOMA-IR, calculated homeostasis model assessment of insulin resistance.

We next examined glucose metabolism in HFD-fed *BAFF*^−/−^ mice; results revealed lower fasting blood glucose and plasma insulin concentrations, as well as lower calculated homeostasis model assessment of insulin resistance indices relative to those in HFD-fed WT mice (Fig. 2A). The glucose levels at random times were also lower in HFD-fed *BAFF*^−/−^ mice than in HFD-fed WT mice (WT: 271 ± 73 mg/dL; *BAFF*^−/−^: 213 ± 42 mg/dL; *n* = 10 /group; *P* < 0.05). Glucose- and insulin-tolerance tests revealed that HFD-fed *BAFF*^−/−^ mice displayed better glucose tolerance and insulin sensitivity than HFD-fed WT mice (Fig. 2B,C). Glucose- and insulin-tolerance did not differ between ND-fed WT and *BAFF*^−/−^ mice (Supplementary Fig. S2). These data indicated that HFD-fed *BAFF*^−/−^ mice displayed significantly improved glucose tolerance as compared with HFD-fed WT mice, despite increased total body and VAT weights.

### Adipose-tissue inflammation is reduced in HFD-fed *BAFF*^−/−^ mice

**Figure 3:**
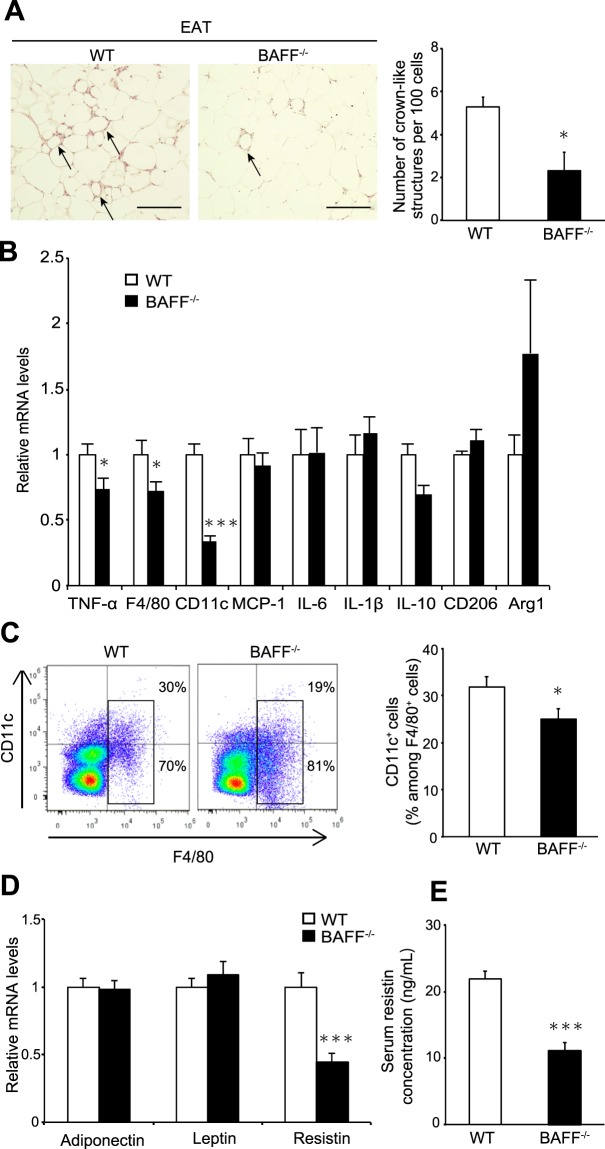
Adipose tissue inflammation is alleviated in *BAFF*^−/−^ mice. (**A**) Representative H&E staining of EAT (left). The arrows point to the CLSs formed by macrophage aggregation. Quantitative measurement of CLSs (right) (scale bar, 200 μm; images from six different fields; *n* = 5/group). (**B**,**D**) Relative mRNA levels of the indicated genes in the EAT (*n* = 12/group). (**C**) Representative flow cytometry plots showing frequencies of CD11c^+^ F4/80^+^ macrophages among stromal cells isolated from EATs from HFD-fed *BAFF*^−/−^ and WT mice (left). Quantification of Cd11c^+^ F4/80^+^ cells isolated from EAT (right) (*n* = 9–12/group). (**E**) Serum resistin concentrations (*n* = 14, 15/group). For all bar plots, data are expressed as the means ± SEM. *P < 0.05 and ***P < 0.001 (Mann–Whitney *U* test). BAFF, B cell-activating factor; WT, wild-type; H&E, haematoxylin and eosin; CLS, crown-like structure; HFD, high-fat diet; EAT, epididymal adipose tissue.

Increased adipose-tissue inflammation is considered a critical determinant of insulin resistance. We observed that the number of crown-like structures (CLSs), which are sites of M1 macrophage aggregation in obese adipose tissue, was significantly reduced in epididymal adipose tissue (EAT) from HFD-fed *BAFF*^−/−^ mice as compared with that in HFD-fed WT mice (Fig. 3A). The expression of *TNF-α* and macrophage-specific markers, such as *F4/80* and *CD11c*, was significantly lower in EAT from *BAFF*^−/−^ mice than in WT mice after HFD feeding (Fig. 3B). However, mRNA levels of M2-like macrophage markers, such as *CD206* and arginase 1, did not differ between the two groups (Fig. 3B). Flow cytometric analysis revealed that the proportion of F4/80^+^ CD11c^+^ M1-like macrophages isolated from the stromal vascular fraction (SVF) of EAT from HFD-fed *BAFF*^−/−^ mice was significantly lower than that in HFD-fed WT mice (Fig. 3C). Furthermore, mRNA levels of *TNF-α* and *resistin*, which plays a role in the development of insulin resistance, were significantly lower in EAT from HFD-fed *BAFF*^−/−^ mice than in EAT from HFD-fed WT mice (Fig. 3B,D), although adiponectin levels did not differ between the two groups. We also observed that serum resistin concentrations were significantly lower in HFD-fed *BAFF*^−/−^ mice as compared with those in HFD-fed WT mice (Fig. 3E) and that serum TNF-α levels were undetectable in both groups. The malondialdehyde (MDA) adduct protein levels, which serve as an indicator of lipid peroxidation and oxidative stress, in the EAT were not different between two groups (Supplementary Fig. 3A). These data indicated that BAFF deficiency was associated with decreases in HFD-induced adipose-tissue inflammation via reduced proinflammatory cytokine levels.

### Adipose-tissue fibrosis is reduced in HFD-fed *BAFF*^−/−^ mice

**Figure 4:**
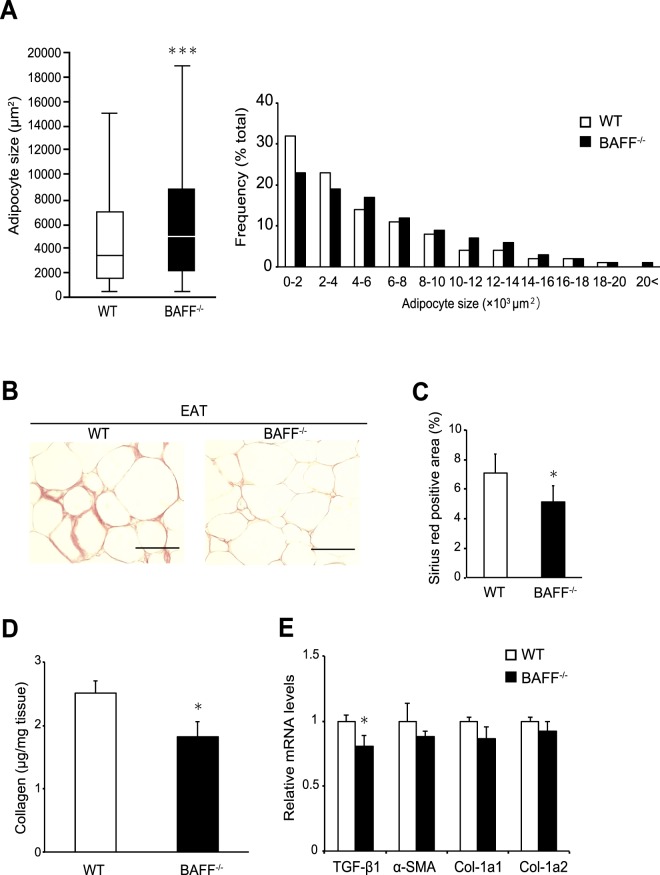
Interstitial fibrosis in white adipose tissue is reduced in *BAFF*^−/−^ mice. (**A**) The median adipocyte size (left) and the distribution of adipocyte size (right) in EAT from HFD-fed *BAFF*^−/−^ and WT mice. Representative (**B**) Sirius red staining and (**C**) quantification of Sirius red-positive areas of EAT (scale bar, 100 μm; images from 10 different fields; *n* = 6/group). (**D**) Total collagen contents in EAT (*n* = 9–13/group). (**E**) Expression of genes related to fibrosis in EAT (*n* = 14–15/group). For all bar plots, data are expressed as the mean ± SEM. *P < 0.05, **P < 0.01, and ***P < 0.001 (Mann-Whitney *U* test). BAFF, B cell-activating factor; HFD, high-fat diet; WT, wild-type; EAT, epididymal adipose tissue; TGF, transforming growth factor; SMA, smooth muscle actin; Col, Collagen.

Adipose-tissue expansion is often associated with adipose-tissue remodelling. In mouse models of abnormal collagen accumulation, rigidity related to developmental fibrosis limits adipose-tissue expansion^13^. Consistent with the observed increase in VAT weight, histological analysis revealed that the adipocyte cell size in EAT was increased in *BAFF*^−/−^ mice as compared with that in EAT from WT mice fed an HFD for 24 weeks (Fig. 4A). By contrast, Sirius red staining revealed extensive interstitial fibrosis of EAT from WT mice, which was significantly reduced in *BAFF*^−/−^ mice (Fig. 4B,C). Additionally, we confirmed that the total collagen content in EAT from *BAFF*^−/−^ mice was significantly lower than that in EAT from WT mice (Fig. 4D). The mRNA expression of transforming growth factor (*TGF*)*-β1* was lower in EAT from *BAFF*^−/−^ mice relative to levels in EAT from WT mice, whereas the α-smooth muscle actin and collagen type 1 mRNA levels did not differ between the two groups (Fig. 4E). These results indicated that a reduction in interstitial fibrosis in EAT from HFD-fed *BAFF*^−/−^ mice may contribute to adipocyte expansion.

### Hepatic steatosis is attenuated in *BAFF*^−/−^ mice fed an HFD

**Figure 5:**
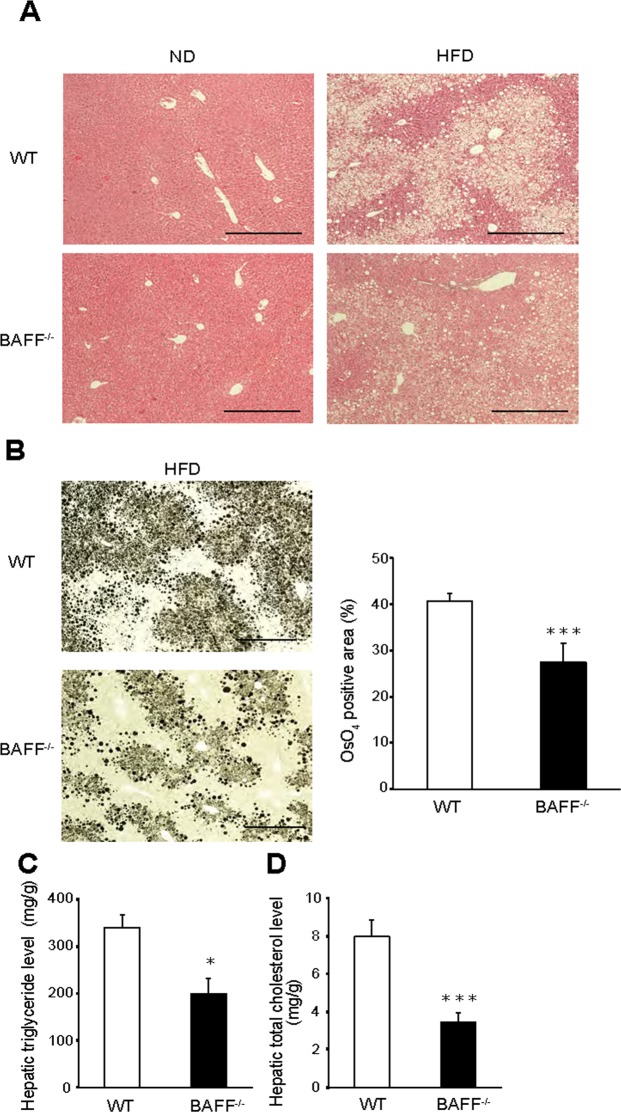
Hepatic steatosis is ameliorated in HFD-fed *BAFF*^−/−^ mice. (**A**) Representative H&E staining of livers from mice after 24 weeks of ND or HFD consumption (scale bar, 500 μm). (**B**) Representative OsO_4_ staining (left) and quantification of the OsO_4_-positive areas (right) of livers (scale bar, 500 μm; images from eight different fields; *n* = 8/group). (**C**) Hepatic TG and (**D**) total cholesterol levels (*n* = 5/group). For all bar plots, data are expressed as the mean ± SEM, and significance was determined by the Mann–Whitney *U* test. *P < 0.05 and ***P < 0.001. BAFF, B cell-activating factor; WT, wild-type; ND, normal diet; HFD, high-fat diet; TG, triglyceride.

We then investigated whether BAFF deficiency influenced the development of fatty liver. Histological examination revealed that hepatic fat accumulation was dramatically lower in HFD-fed *BAFF*^−/−^ mice than in HFD-fed WT mice (Fig. 5A,B), which was consistent with the lower liver weight observed in *BAFF*^−/−^ mice. Furthermore, liver TG and cholesterol levels were significantly lower in HFD-fed *BAFF*^−/−^ mice relative to those in HFD-fed WT mice (*P* < 0.01; Fig. 5C,D), although serum alanine transaminase (ALT) levels did not differ between the two groups (WT: 111 ± 54 IU/L; *BAFF*^−/−^: 111 ± 35 IU/L; *n* = 7/group; *P = *0.81). Additionally, histological examination revealed no or mild inflammation in the livers of both groups (Fig. 5A).

**Figure 6:**
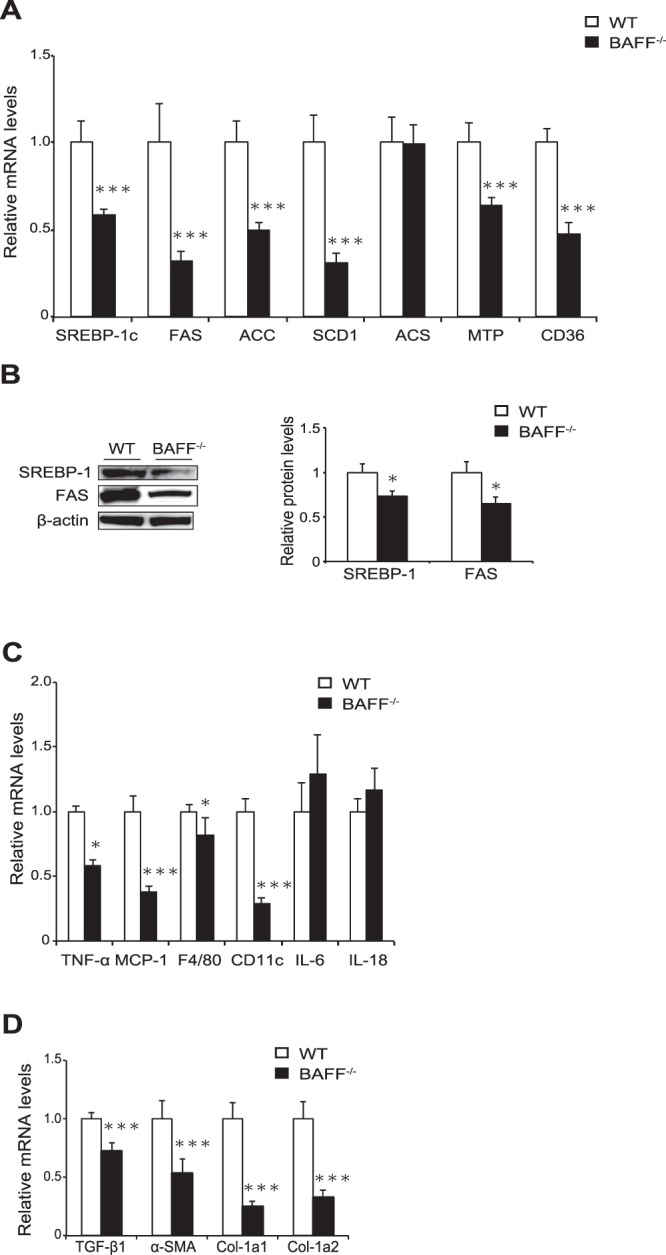
The expression of genes related to steatosis is downregulated in the livers of *BAFF*^−/−^ mice. (**A**,**C**,**D**) The expression of genes related to lipogenesis (**A**), inflammation (**C**), and fibrosis (**D**) in mouse livers after 24 weeks of HFD consumption (*n* = 9–14/group). (**B**) The expression of proteins related to lipogenesis (*n* = 4/group). Original gel documents of western blot were shown in Supplementary Fig. S4. Protein levels were normalized to those of β-actin, and those of WT mice were set at 1. For all bar plots, data are expressed as the mean ± SEM, and significance was determined by Mann–Whitney *U* test. *P < 0.05 and ***P < 0.001. BAFF, B cell-activating factor; WT, wild-type; HFD, high-fat diet; FAS, fatty acid synthase; SREBP1, sterol regulatory element-binding protein; ACC, acetyl-CoA carboxylase; SCD, stearoyl-CoA desaturase; ACS, acetyl-CoA synthetase; MTP, microsomal triglyceride transfer protein; TNF, tumour necrosis factor.

To investigate mechanisms underlying the attenuation of hepatic steatosis in HFD-fed *BAFF*^−/−^ mice, we analysed the expression of genes related to lipid metabolism in the livers of both groups. The expression of *CD36*, which is related to fatty acid uptake and transport, was significantly downregulated in the livers of HFD-fed *BAFF*^−/−^ mice relative to levels in HFD-fed WT mice, as were levels of sterol regulatory element-binding protein 1c (*SREBP-1c)* and fatty acid synthase (*FAS)*, which are related to lipogenesis (Fig. 6A). We confirmed that the SREBP-1c and FAS protein levels were also lower in the livers of *BAFF*^−/−^ mice (Fig. 6B). Additionally, levels of *TNF-α*, monocyte chemoattractant protein-1, and macrophage markers such as *F4/80* and *CD11c* were significantly lower in the livers of *BAFF*^−/−^ mice than in those of WT mice (Fig. 6C), although histologic analysis revealed that inflammatory cell infiltration did not differ between groups according to histologic analysis (Fig. 5A). The MDA adduct protein levels in the livers did not differ between the two groups (Supplementary Fig. 3B). These data suggested that BAFF deficiency prevented hepatic steatosis by decreasing *de novo* lipogenesis in the liver as well as fatty acid influx from EAT.

### BAFF deficiency decreases lipid synthesis in hepatocytes

**Figure 7:**
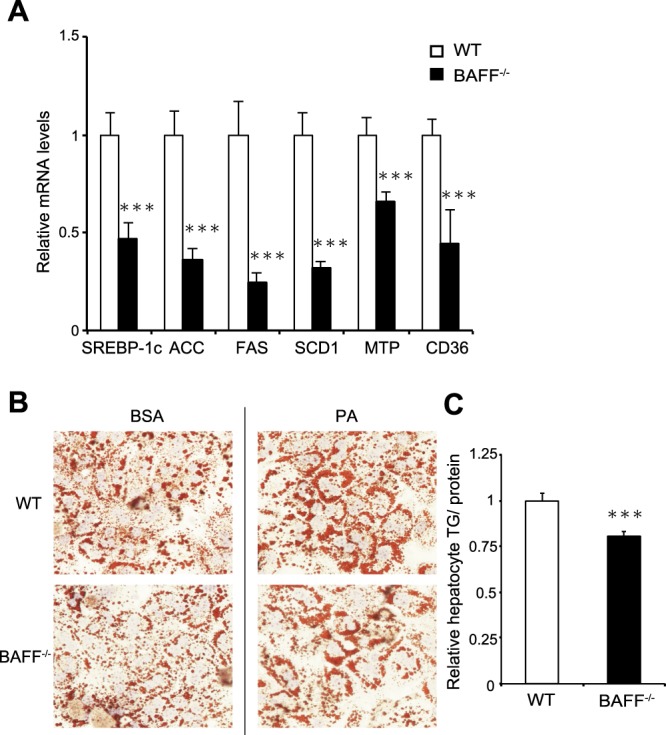
Hepatocyte fat deposition is reduced in *BAFF*^−/−^ mice. (**A**) The expression of genes related to lipogenesis in primary hepatocytes from HFD-fed *BAFF*^−/−^ and WT mice. (**B**) Hepatocyte fat deposition. Representative Sudan III staining of primary hepatocytes from *BAFF*^−/−^ and WT mice exposed to BSA or PA (left). Quantification of TG in primary hepatocytes exposed to PA (right) (*n* = 8/group). For all bar plots, data are expressed as the mean ± SEM. ***P < 0.001 (Mann–Whitney *U* test). BAFF, B cell-activating factor; WT, wild-type; FAS, Fatty acid synthase; SREBP1, sterol regulatory element-binding protein; ACC, acetyl-CoA carboxylase; SCD, stearoyl-CoA desaturase; MTP, microsomal triglyceride transfer protein; BSA, bovine serum albumin; PA, palmitate, TG; triglyceride.

We next evaluated the expression of genes related to lipid metabolism using primary cultured hepatocytes from HFD-fed mice. Consistent with the results obtained from whole livers, the expression of genes related to lipid synthesis was significantly downregulated in hepatocytes from *BAFF*^−/−^ mice as compared with that in hepatocytes from WT mice (Fig. 7A).

Furthermore, we analysed the role of BAFF in lipid accumulation in an *in vitro* model of hepatic steatosis. Primary cultured hepatocytes were exposed to palmitate *in vitro*. As shown in Fig. 7B, fat accumulation was attenuated to a greater degree in primary cultured hepatocytes from *BAFF*^−/−^ mice than in those from WT mice. These results indicated that BAFF deficiency profoundly attenuated lipid accumulation in the hepatocytes of HFD-fed mice.

## Discussion

We previously reported that BAFF impairs insulin sensitivity and is associated with NAFLD severity^9,11^, indicating that this molecule is an important factor in glucose and lipid metabolism. In the present study, we demonstrated that BAFF deficiency has beneficial effects on metabolic dysfunction in diet-induced obesity models. First, as expected, HFD-fed *BAFF*^−/−^ mice showed lower fasting glucose levels than HFD-fed WT mice (Fig. 2A). Additionally, HFD-fed *BAFF*^−/−^ mice showed better glucose tolerance and insulin sensitivity after insulin challenge than HFD-fed WT mice (Fig. 2B,C). Moreover, HFD-fed *BAFF*^−/−^ mice displayed marked improvements in hepatic steatosis as compared with that observed in HFD-fed WT mice (Fig. 5), despite a lack of body weight reduction. Both insulin and glucose stimulate the expression of *SREBP-1*, a major transcription factor that positively regulates *de novo* lipogenic enzymes in the liver^14^. Yahagi *et al*.^15^ reported that the absence of SREBP-1 ameliorates fatty liver development, but not obesity or insulin resistance, in leptin-deficient ob/ob mice. In the present study, we observed that BAFF deficiency attenuated hyperglycaemia in HFD-fed mice and was associated with the downregulation of genes related to *de novo* lipogenesis, including *SREBP-1c* and *FAS*, in the liver (Fig. 6A,B). We also confirmed that the expression of genes related to lipid synthesis was significantly downregulated in primary cultured hepatocytes obtained from HFD-fed *BAFF*^−/−^ mice (Fig. 7A) and that BAFF deficiency alleviated palmitate-induced fat deposition in primary cultured hepatocytes *in vitro* (Fig. 7B). These data indicated that a decrease in *de novo* lipogenesis directly contributed to the attenuation of hepatic steatosis in HFD-fed *BAFF*^−/−^ mice.

In addition to *de novo* lipogenesis, liver fatty acids are derived from VAT. Obesity induces chronic low-grade inflammation in VAT^16^, which leads to morphological and functional changes, including alterations in VAT-resident immune-cell profiles, dynamic remodelling of the extracellular matrix (ECM), and altered production of adipokines^17^. Moreover, we previously reported that BAFF was preferentially expressed in VAT and inhibited insulin-signalling pathways in adipocytes^9^. As expected, in the present study, we demonstrated that BAFF deficiency reduced the accumulation of proinflammatory CD11c^+^ macrophages and CLS formation in VAT from HFD-fed mice (Fig. 3A,C). Additionally, levels of resistin, whose expression is induced during adipogenesis to interfere with multiple steps in the insulin-signalling cascade, were significantly lower in HFD-fed *BAFF*^−/−^ mice than in HFD-fed WT mice (Fig. 3D,E). This finding is consistent with results from our previous report showing that BAFF induced resistin expression in adipocytes *in vitro* and *in vivo*^9^. These results suggest that BAFF may drive insulin resistance related to adipokine production, as well as suppress the infiltration of proinflammatory macrophages into VAT.

In this study, we observed that HFD-fed *BAFF*^−/−^ mice displayed significant increases in EAT weight and adipocyte size (Figs 1 and 4) but decreases in liver weight and hepatic fat accumulation as compared with HFD-fed WT mice (Figs 1 and 5). A previous study demonstrated that hepatic steatosis is associated with the loss of epididymal fat mass in HFD-fed mice^18^. Moreover, in humans, loss of adipocyte mass (lipodystrophy) is associated with hepatomegaly due to steatosis^19^. These observations are consistent with our findings. In addition to insulin resistance and proinflammatory conditions, adipose-tissue fibrosis may be involved in this process. A previous study suggested that excessive accumulation of ECM proteins (i.e., collagen) is a key feature of adipose-tissue dysfunction during obesity^20^. Further, several groups have reported that fibrosis of VAT limits lipid-storage capacity by inhibiting adipocyte hypertrophy, which plays a role in ectopic lipid accumulation in the liver^21,22^. Divoux *et al*.^23^ reported that adipose-tissue fibrosis is negatively correlated with adipocyte diameter in human adipose tissue. Moreover, increased interstitial fibrosis decreases ECM flexibility, reduces tissue plasticity, and limits adipose-tissue expansion^24^, leading to an increased frequency of adipocyte cell death and enhanced inflammatory response. TGF-β signalling contributes to ECM deposition, specifically in VAT^25^. The reduced *TGF-β1* expression in VAT from HFD-fed *BAFF*^−/−^ mice (Fig. 4), as observed in the present study, may support this process. VAT expansion in the relative absence of fibrosis and inflammation maintains metabolic homeostasis in response to an energy surfeit^26^ and represents a hallmark of metabolically “healthy” obesity^27^. These data suggest that *BAFF*^−/−^ mice are protected against obesity-induced adipose-tissue fibrosis and exhibit increased lipid-storage capacity, which may reduce FFA efflux from the VAT to the liver. Recent studies indicate that adipose oxidative stress in the adipose tissue is associated with metabolic disorders and obesity, including healthy adipose tissue expansion and ectopic lipid accumulation^28^. However, oxidative stress levels, as estimated by the levels of MDA adducts, in the VAT were not different between HFD-fed *BAFF*^−/−^ mice and HFD-fed WT mice (Supplementary Fig. 3A).The reasons for this discrepancy are not known; further studies are necessary in this regard.

Here, we demonstrated for the first time a role for BAFF in hepatic steatosis using *BAFF*^−/−^ mice. Previously, Kim *et al*.^29,30^ reported that BAFF depletion ameliorated glucose intolerance and resulted in increased body weight in HFD-fed mice, which is consistent with our observations. However, they^30^ showed that *BAFF*^−/−^ mice did not exhibit an elevated fat mass in the EAT as compared with WT mice fed with an HFD for 10 weeks. Additionally, liver weight did not differ between the two groups, although they did not investigate liver histology. This discrepancy may be caused by the different protocols used in the two studies. Strissel *et al*.^18^ reported that EAT weight and hepatic steatosis were inversely correlated after 12 weeks of HFD feeding, which may be related to adipocyte death and adipose-tissue remodelling.

The phenotype of BAFF-R-deficient mice is similar to that of *BAFF*^−/−^ mice^31^. Both types of mice exhibit a reduced number of mature B cells and impaired antigen-dependent antibody responses. Additionally, both *BAFF-R*^−/−^ and *BAFF*^−/−^ mice exhibit attenuated inflammation in VAT and improved insulin resistance^12^. However, HFD-fed *BAFF-R*^−/−^ mice displayed exacerbated hepatic steatosis along with elevated expression of genes related to *de novo* lipogenesis^12^. The reasons for this discrepancy between *BAFF-R*^−/−^ and *BAFF*^−/−^ mice are unclear; however, the serum BAFF concentrations in *BAFF-R*^−/−^ mice were approximately threefold higher than those in WT mice (data not shown). In our preliminary study, we measured serum BAFF concentrations in patients with histologically diagnosed NAFLD (*n* = 65) and found that the prevalence of severe steatosis (>66%) was significantly higher among patients with higher BAFF concentrations than among those with lower BAFF concentrations (data not shown). This may support the idea that BAFF plays a role in exacerbating hepatic steatosis. We analysed the expression levels of three previously identified BAFF receptors (BAFF-R, B cell-maturation antigen, and transmembrane activator and calcium modulating cyclophilin-ligand interactor) and found that BAFF-R may represent the main functional receptor in the mouse liver^12^. However, the roles of other receptors, including those that remain unidentified, cannot be disregarded. Furthermore, recent evidence has indicated that B cells contribute to the development and promotion of insulin resistance^32^, adipose-tissue dysfunction^33^, and NAFLD^34^. In the present study, we primarily focused on the metabolic aspects of BAFF in VAT and the liver; however, BAFF also plays an important role in maintaining a healthy immune system^8^. Therefore, future studies may be necessary to investigate the immunological role(s) of BAFF and B-cell compartments.

In summary, we demonstrated that BAFF depletion improved HFD-induced insulin resistance and liver steatosis via suppression of VAT inflammation and fibrosis, subsequently leading to beneficial VAT expansion. Our data suggested that targeting BAFF might be beneficial for treating obesity-related NAFLD and diabetes.

## Methods

### Animals

All methods were carried out in accordance with the guidelines and regulations of Ehime University (Ehime, Japan), and all experimental protocol was approved by Ehime University Animal Research (No. 05TI70-16).

Male C57BL/6J WT mice and B6.129S2-Tnfsf13b^tm1Msc^/J (*BAFF*^−/−^) mice in C57BL/6J background were purchased from CLEA Japan (Tokyo, Japan) and the Jackson Laboratory (Bar Harbor, ME, USA), respectively. They were maintained in a temperature-, humidity-, and light-controlled room (12-h light/dark cycles), allowed free access to water, and fed an ND (13% fat, 26% protein, and 60% carbohydrates; 360 kcal/100 g; Oriental Yeast, Tokyo, Japan). Six-week-old animals were fed *ad libitum* with ND or HFD (D12492; 60% fat, 20% protein, and 20% carbohydrates; 520 kcal/100 g; Research Diets, New Brunswick, NJ, USA).

Serum was extracted after 15 h of fasting and stored at −80 °C. In some experiments, serum was extracted at random times. Serum TG and ALT levels were measured using a Hitachi 7180 Autoanalyzer (Hitachi, Ltd., Tokyo, Japan). The liver and EAT were harvested, submerged in RNA-later (Life Technologies, Carlsbad, CA, USA) overnight, and stored at −20 °C until use.

### Glucose- and insulin-tolerance tests

Glucose-tolerance tests were performed after a 16-h fast. Blood glucose concentrations were measured by a blood glucose test meter (Antisense III; HORIBA Medical, Kyoto, Japan) at 0, 15, 30, 60, 90, and 120 min after intraperitoneal injection of glucose (1.5 mg/g body weight). Insulin sensitivity was assessed using an insulin-tolerance test. After 6 h of fasting, insulin (1 U/kg body weight; Eli Lilly, Indianapolis, IN, USA) was administered intraperitoneally, and blood samples were drawn from the tail vein at 0, 30, 60, 90, and 120 min after administration. Plasma insulin levels were measured with an ELISA kit (Morinaga Institute of Biological Science, Kanagawa, Japan).

### Histological and morphometric analysis

Liver tissue and EAT were fixed with neutral-buffered formalin and embedded in paraffin. Sections (3-µm-thick) were stained with haematoxylin and eosin (H&E) or Sirius red, and adipocyte size and number in the EAT were measured digitally in H&E sections (10×) using ImageJ software (6 sections per animal, *n* = 5 animals/group; National Institutes of Health, Bethesda, MD, USA). The CLS density of EAT was obtained by counting the total numbers of CLSs and adipocytes per section. For the measurement of fibrosis, Sirius red-positive areas were measured digitally in histological light-microscopy images (10×; 10 sections per animal, *n* = 6 animals/group). To evaluate the degree of fat accumulation, livers were stained with OsO_4_. Briefly, fixed livers were fixed again with buffer containing 2% OsO_4_, 5% potassium dichromate, and acetic acid for 24 h, and OsO_4_-positive areas were measured digitally using ImageJ software (6 sections per animal, *n* = 8 animals/group; National Institutes of Health).

### Isolation of SVF and flow cytometric analysis

EAT was collected and cut into small pieces. After filtering through a 40-μm strainer, tissues were centrifuged and resuspended in RPMI(−) medium. The dissociated SVF was washed twice with PBS, incubated for 10 min in erythrocyte-lysing buffer, and washed again with PBS. Cells were stained with the following antibodies and incubated for 30 min at 4 °C in the dark: CD11c (BioLegend, San Diego, CA, USA) and F4/80 (BD Biosciences, Franklin Lakes, NJ, USA). Staining was analysed by fluorescence-assisted cell sorting (Galios; Beckman Coulter, Tokyo, Japan) using FlowJo 7.6.3. software (Tree Star, Ashland, OR, USA).

### Quantitative real-time PCR

RNA was extracted from livers and EAT using an RNeasy Plus Mini Kit (Qiagen, Hilden, Germany) and an RNeasy Plus Lipid Kit (Qiagen), respectively. Reverse-transcription reactions were performed using SYBR Green I (Roche Diagnostics, Basel, Switzerland) on a LightCycler 480II (Roche Diagnostics). Primer sequences and annealing temperatures are provided in Supplementary Table S1. Gene-expression data were normalized with the housekeeping gene encoding hypoxanthine phosphoribosyltransferase 1 and expressed as a ratio to values obtained for WT mice.

### ELISA

Serum concentrations of BAFF and resistin were determined using ELISA (MBLYS0 for BAFF and MRSN00 for resistin; R&D Systems, Minneapolis, MN, USA). The MDA adduct levels in the liver and EAT were estimated using a commercially available ELISA kit (Oxiselect MDA Adduct Competitive ELISA kit; Cell Biolabs, San Diego, CA, USA), according to the manufacturer’s protocol.

### Collagen content in adipose tissue

Total collagen content in adipose tissue was measured using a commercially available kit (QuickZyme total collagen assay; QuickZyme Biosciences, Leiden, Netherlands).

### Measurement of hepatic TG and cholesterol

Hepatic TG and cholesterol levels were measured at Skylight Biotech (Akita, Japan) using the Folch technique with Cholestest TG and Cholestest CHO kits (Sekisui Medical, Tokyo, Japan), respectively.

### Western blotting analysis

Total liver and isolated primary hepatocyte lysates were prepared using radioimmunoprecipitation assay buffer with a protease-inhibitor cocktail (Sigma-Aldrich, St. Louis, MO, USA). Protein (30 μg) was placed in the wells of 4–12% Bis-Tris Gels (Invitrogen, Carlsbad, CA, USA) and resolved. The products were then blotted onto a polyvinylidene difluoride membrane using NuPage transfer buffer and XCell SureLock (both from Invitrogen) and incubated with the following specific antibodies at 4 °C overnight: FAS (1:1000; #3180; Cell Signaling Technology, Danvers, MA, USA), SREBP-1 (1:1000; AB28481; Abcam, Cambridge, UK), and β-actin (1:3000; MAB1501; Millipore, Billerica, MA, USA).

### Isolation of primary cultured hepatocytes

Primary cultured hepatocytes were isolated by liver perfusion. Briefly, after digestion with Liver Digest Medium (Life Technologies), the liver was excised, minced, filtered, and centrifuged. We washed the samples twice with Williams’ Medium E (Gibco; Life Technologies, Gaithersburg, MD, USA) supplemented with 10% foetal bovine serum (FBS), 1% penicillin–streptomycin, insulin, 1% l-glutamine, and 1.5% HEPES. For mRNA-expression analysis, hepatocytes were used before attachment. To assess fat deposition, after attachment, primary cultured hepatocytes were cultured with Williams’ Medium E supplemented with 10% FBS, 1% penicillin–streptomycin, 1% l-glutamine, and 1.5% HEPES added to bovine serum albumin (BSA) or palmitate (0.3 mM) for 18 h.

### Measurement of hepatocyte fat deposition

Cells were stained with Sudan III, and additional cells were harvested and subjected to a chemical assay to quantify intracellular levels of TGs using a Triglyceride E-Test (Wako Pure Chemical Company, Osaka, Japan) using the Bligh–Dyer procedure.

### Statistical analysis

Data were analysed using JMP version 11.2.0 software (SAS Institute, Cary, NC, USA), and values are presented as the mean ± SEM or SD. Normally distributed and skewed data were analysed using Student’s *t* tests and Mann–Whitney *U* tests, respectively. Differences were considered statistically significant at *P* < 0.05.

## Supplementary information

Supplementary Information

## Data Availability

The datasets generated during and/or analysed during the current study are available from the corresponding author on reasonable request.
