# Supplementary material for: Depletion of B cell-activating factor attenuates hepatic fat accumulation in a murine model of nonalcoholic fatty liver disease
Source: Sci Rep. 2019 Jan 30;9:977. doi: 10.1038/s41598-018-37403-y (PMC6353938; doi:10.1038/s41598-018-37403-y)
Supplement: Supplementary file 1 — Supplementary Information [file 41598_2018_37403_MOESM1_ESM.pdf]

Supplementary Information

Depletion of B cell-activating factor attenuates hepatic fat accumulation in a murine model of nonalcoholic fatty liver disease

Yoshiko Nakamura, Masanori Abe, Keitarou Kawasaki, Teruki Miyake, Takao Watanabe, Osamu Yoshida, Masashi Hirooka, Bunzo Matsuura, Yoichi Hiasa

Table S1. Oligonucleotide sequences and annealing temperatures for quantitative real-time PCR.

Gene	GenBank Accession No.	Sequence (5′-forward-3′ 5′-reverse-3′)	Location	Annealing Temperature (°C)
HPRT1	NM_013556	tcctcctcagaccgcttt<br>cctggttcatcatcgctaatc	nt104–122<br>nt173–193	60
F4/80	NM_010130	agtacgatgtggggcttttg<br>ccccatctgtacatcccact	nt35–54<br>nt77–96	60
CD11c	NM_021334	atggagcctcaagacaggac<br>ggatctgggatgctgaaatc	nt1725–1744<br>nt1768–1787	60
TNF-α	NM_013693	tcttctcattcctgcttgtgg<br>ggtctgggccatagaactga	nt260–280<br>nt368–387	60
CD36	NM_016741	cgtctacccacccaacga<br>agaaacagaggcgcacca	nt1148–1165<br>nt1220–1237	65
SREBP-1c	ENSMUST0000 0144942	catggattgcacatttgaaga<br>cgggaagtcactgtcttggt	nt111–131<br>nt152–171	65
FAS	NM_007988	gctgctgttggaagtcagc<br>agtgttcgttcctcggagtg	nt767–785<br>nt823–842	70
ACC	NM_133360	gcgtcgggtagatccagtt<br>ctcagtggggcttagctctg	nt6434–6452<br>nt6484–6503	60
SCD-1	NM_009127	ttccctcctgcaagctctac<br>cagagcgctggtcatgtagt	nt566–585<br>nt608–627	60
ACS	NM_007981	aaagatggctggttacacacg<br>cgataatcttcaaggtgccatt	nt1818–1838<br>nt1863–1884	57
CD36	NM_001159555	ttgtacctatactgtggctaaatgaga<br>cttgtgttttgaacatttctgctt	nt1689–1715<br>nt1737–1760	57
MTP	NM_008642	tgtcagaatgaaggctgcaa<br>agtcctcccaggatcagctt	nt1395–1414<br>nt1440–1459	60
TGF-β1	NM_011577	tggagcaacatgtggaactc<br>cagcagccggttaccaag	nt1358–1377<br>nt1411–1428	60
IL-6	NM_031168	acaaccacggccttccctactt<br>cacgatttcccagagaacatgtg	nt92–113<br>nt198–220	63
IL-1β	NM_008361	ttgacggaccccaaaagat<br>agctggatgctctcatcagg	nt142–160<br>nt195–214	60
IL-10	NM_010548	ggttgccaagccttatcgga<br>acctgctccactgccttgct	nt302–321<br>nt473–492	63
IL-18	NM_008360	gacaacacgctttactttatacctga<br>cagtgaagtcggccaaagtt	nt73–84<br>nt99–112	70
MCP-1	NM_011333	catccacgtgttggctca<br>gatcatcttgctggtgaatgagt	nt139–56<br>nt192–214	70

Resistin	ENSMUST0000 0012849	tgccagtgtgcaaggataga<br>tggaaaccacgctcacttc	nt367–386<br>nt437–455	70
Adiponectin	NM_009605	ggagagaaaggagatgcaggt<br>ctttcctgccaggggttc	nt319–339<br>nt410–427	67
α-SMA	NM_007392	cccacccagagtggagaa<br>acatagctggagcagcgtct	nt9–26<br>nt55–74	65
Col-1a1	NM_007742	caggcaagcctggtgaac<br>aacctctctcgcctcttgc	nt2030–2047<br>nt2089–2107	65
Col-1a2	NM_007743	gctgctcagtattctgacaaagg<br>cctctgggtcccattaaacc	nt393–415<br>nt441–460	65
CD206	NM_008625.2	ggtctatggaaccacggatg<br>tgcattgcccagtaaggagt	nt519–538<br>nt569–588	63
Arg1	NM_007482.3	gtctgtggggaaagccaat<br>cttccaactgccagactgtg	nt315–333<br>nt403–422	60

HPRT1: hypoxanthine phosphoribosyltransferase 1, TNF: tumour necrosis factor; SREBP: sterol regulatory element-binding protein; FAS: fatty acid synthase; ACC: acetyl-CoA carboxylase; SCD: stearoyl-CoA desaturase; ACS: acyl-CoA synthetase, MTP: microsomal triglyceride transfer protein; TGF: transforming growth factor; IL: interleukin; MCP-1: monocyte chemotactic protein-1, SMA: smooth muscle actin, Col: collagen, Arg1: arginase 1

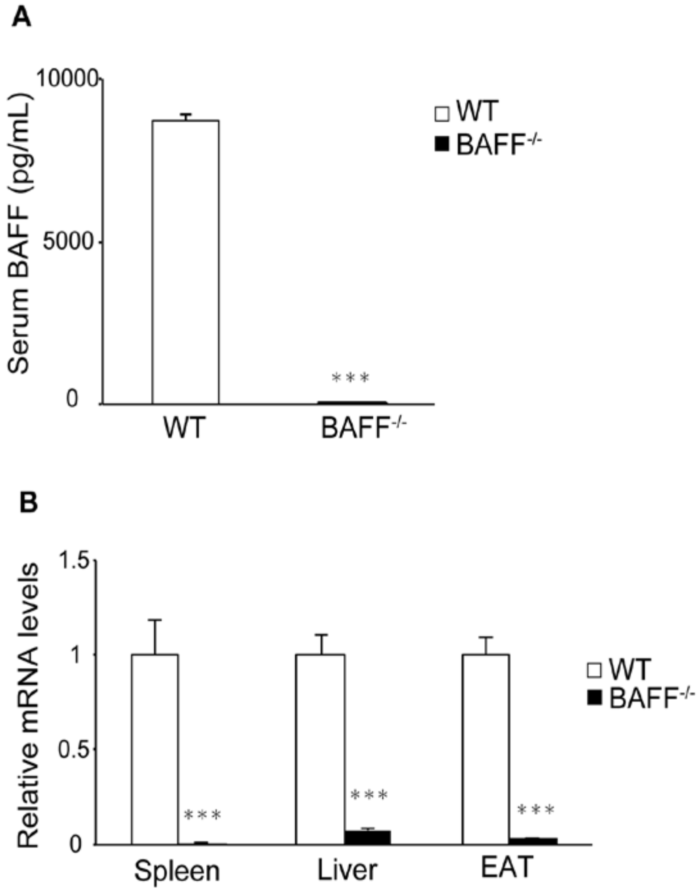

Supplementary Fig. S1. Verification of *BAFF* knockout. *BAFF* knockout was confirmed by (A) measurement of serum BAFF concentrations (n = 6) and (B) real-time PCR for quantitation of *BAFF* levels in samples from spleens, livers, and EAT (n = 8). $BAFF^{-/-}$ and C57BL/6J mice were fed an ND for 18 weeks. Data represent the mean ± SEM. ***P < 0.001, Mann–Whitney U test.

BAFF, B cell-activating factor; EAT, epididymal adipose tissue; ND, normal diet.

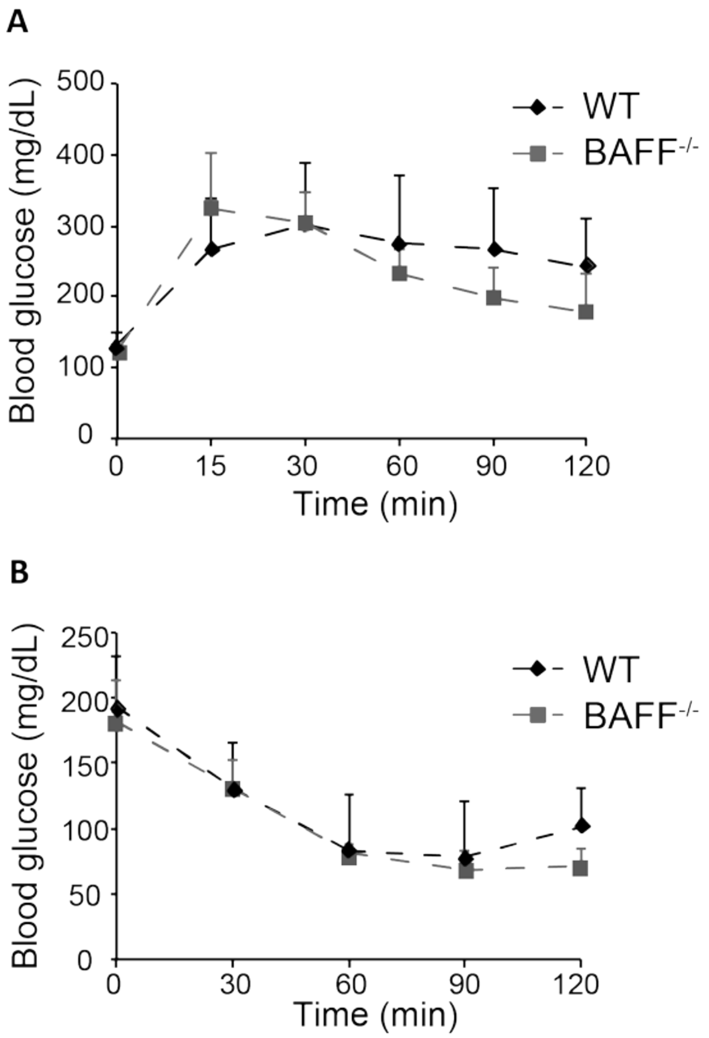

Supplementary Fig S2. Insulin sensitivity in *BAFF*$^{-/-}$ mice fed with an ND

(A) Glucose- and (B) insulin-tolerance tests in ND-fed WT and *BAFF*$^{-/-}$ mice (n = 5/group). For all bar plots, data are expressed as the mean ± SD.

BAFF, B cell-activating factor; ND, normal diet; WT, wild-type

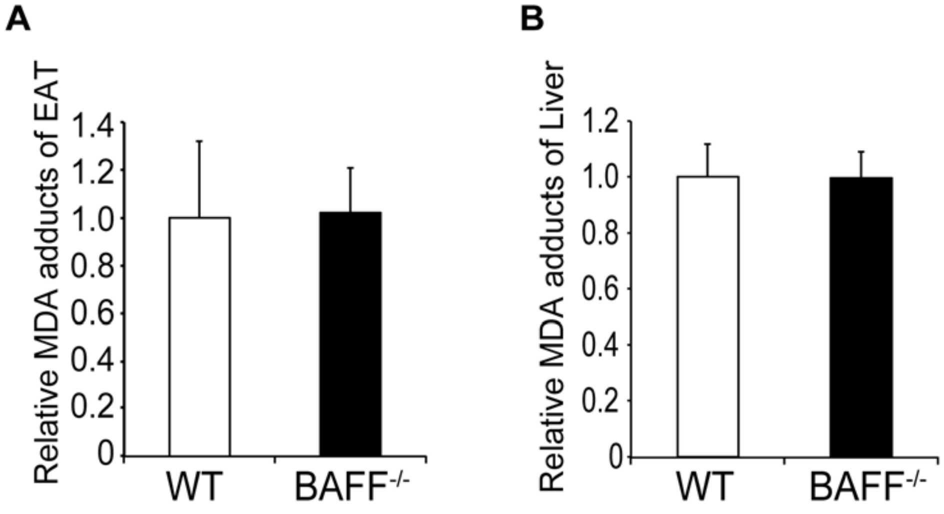

Supplementary Fig. 3 MDA adduct levels in the EAT and liver

MDA adduct levels of lysate of (A) EAT and (B) liver from *BAFF*$^{-/-}$ and WT mice fed with an HFD for 24 weeks, were estimated by ELISA (*n* = 7/group). Data are expressed as the mean ± SD and expressed as a ratio to values obtained for the WT mice.

MDA, malondialdehyde; BAFF, B cell-activating factor; EAT, epididymal adipose tissue; ND, HFD, high-fat diet; WT, wild-type

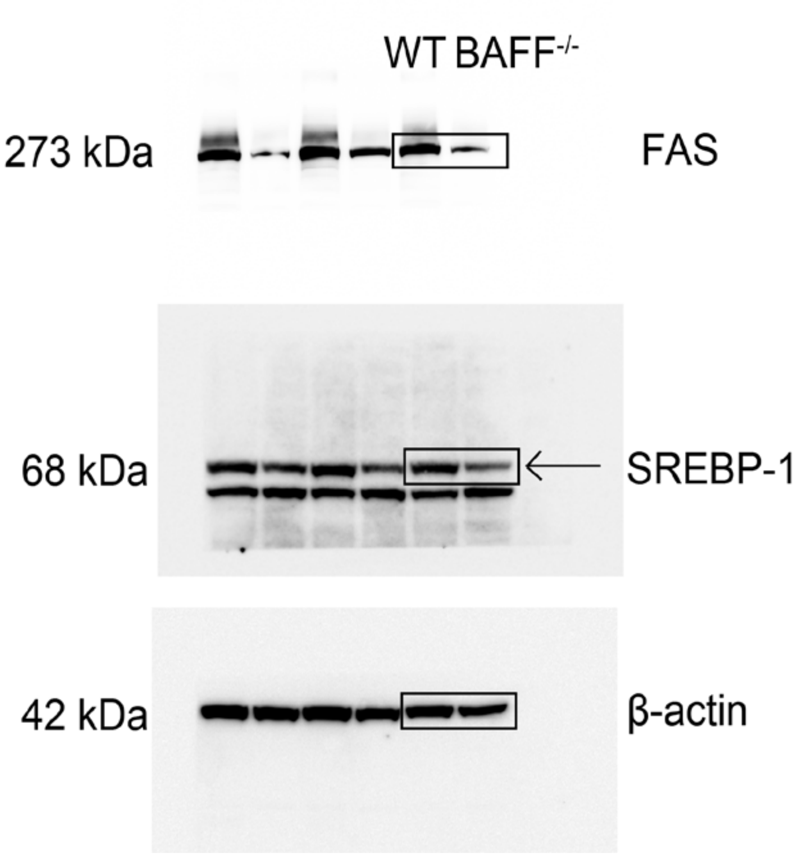

Supplementary Fig. S4. Original blots for Figure 6B.
